# Detection of Anomalous Grapevine Berries Using Variational Autoencoders

**DOI:** 10.3389/fpls.2022.729097

**Published:** 2022-06-01

**Authors:** Miro Miranda, Laura Zabawa, Anna Kicherer, Laurenz Strothmann, Uwe Rascher, Ribana Roscher

**Affiliations:** ^1^Remote Sensing Group, Institute of Geodesy and Geoinformation, University of Bonn, Bonn, Germany; ^2^Institute of Geodesy and Geoinformation, Professorship of Geodesy, University of Bonn, Bonn, Germany; ^3^Julius Kühn-Institut, Institute for Grapevine Breeding Geilweilerhof, Geilweilerhof, Germany; ^4^Institute of Bio- and Geosciences IBG-2, Plant Sciences, Forschungszentrum Jülich, Jülich, Germany; ^5^International AI Future Lab, Technical University of Munich, Munich, Germany

**Keywords:** autoencoder, deep learning, anomaly detection, viticulture, disease detection

## Abstract

Grapevine is one of the economically most important quality crops. The monitoring of the plant performance during the growth period is, therefore, important to ensure a high quality end-product. This includes the observation, detection, and respective reduction of unhealthy berries (physically damaged, or diseased). At harvest, it is not necessary to know the exact cause of the damage, but rather if the damage is apparent or not. Since a manual screening and selection before harvest is time-consuming and expensive, we propose an automatic, image-based machine learning approach, which can lead observers directly to anomalous areas without the need to monitor every plant manually. Specifically, we train a fully convolutional variational autoencoder with a feature perceptual loss on images with healthy berries only and consider image areas with deviations from this model as damaged berries. We use heatmaps which visualize the results of the trained neural network and, therefore, support the decision making for farmers. We compare our method against a convolutional autoencoder that was successfully applied to a similar task and show that our approach outperforms it.

## 1. Introduction

The constant and regular monitoring of plant performance is important in agriculture to ensure efficient and sustainable production and a reduction of yield losses caused, e.g., by diseases or pests. Especially in viticulture, this is a crucial aspect due to the ongoing climate change, which causes more extreme and on average higher temperatures, an increase in water and drought stress, higher CO_2_ amounts in the atmosphere, and changing abundance of pests (Jones, 2007). To ensure a high quality end-product it is important to reduce the number of damaged berries before harvest (Charters and Pettigrew, 2007), in many cases without the need to know the reason for the damage. This, however, is a labor-intensive task that is still mainly carried out by experts in the field during the harvest (Bramley et al., 2005). To ease this process, research focuses on objective and automated approaches for machine-driven high-throughput phenotyping in agriculture (Kamilaris and Prenafeta-Boldú, 2018) and viticulture (Tardaguila et al., 2021). For this purpose, imaging sensors are widely used due to their affordability and their ability to provide a suitable data basis for analysis and interpretation (Kamilaris and Prenafeta-Boldú, 2018; Ma et al., 2019).

Since 2012, especially convolutional neural networks (CNNs) have proven to be a powerful approach, as they can recognize spatial structures in images and capture typical characteristics of objects (Schmidhuber, 2015). The identification and localization of plant diseases using CNNs can be achieved with several task formulations which are mostly trained in a supervised manner (Bah et al., 2018; Kamilaris and Prenafeta-Boldú, 2018; Kaur et al., 2019). This includes classification, object detection, and segmentation, which all require costly annotations. Many studies focus on the detection of diseases on leaves (Khirade and Patil, 2015), e.g., Yadhav et al. (2020) perform a multi-class classification with a shallow CNN to detect diseases. To detect damaged berries, Bömer et al. (2020) propose a CNN which performs a supervised classification to create heatmaps for grape bunches, where the heatmap values are meant to indicate the severity of berry damage.

In contrast to many supervised classification approaches that distinguish between healthy and well-defined diseases and, therefore, require labels for both classes, there are approaches that are fully unsupervised or work only with information about the healthy plants or plant parts. The general idea of these approaches is to learn a representation of healthy samples and define deviations from this representation as an anomaly (Pang et al., 2021). The main advantage is that no manual annotation and labeling of anomalies, such as specific diseases, is required, thus bypassing the expensive collection of these labels and the required guidance of an expert to label them correctly and accurately. It also avoids a full capture of the variability of anomalies, such as all possible plant diseases, that would be necessary to learn a representative classifier.

Recent studies in this field propose the use of autoencoders (AEs), variational autoencoders (VAEs), or generative adversarial networks (GANs), which can be trained on non-anomalous data without defining the characteristics of specific anomalies. Studies such as the one of Picetti et al. (2018) use a convolutional autoencoder (CAE) to detect buried landmines in ground penetrating radar (GPR) observations without making assumptions about the size or shape of the detected objects. As a close-range application, Akçay et al. (2018) use a CAE with three different losses (contextual, encoder, and adversarial loss) to detect anomalies in in-flight luggage. Another prominent example is the detection of anomalies in surveillance videos. The study presented in Zhao et al. (2017) uses CAEs while (Chong and Tay, 2017) use spatio-temporal autoencoder (AE). More examples of CAE to detect anomalies can be found in Chalapathy et al. (2017), Ke et al. (2017), Baur et al. (2019), and Mesquita et al. (2019). Other studies use AE as feature extractors and make use of a subsequent classifier, often Support Vector Machine (SVM). In the context of precision agriculture, Pardede et al. (2018) use a CAE as a feature extractor for an SVM-classification algorithm to detect plant diseases.

An and Cho (2015) proposed an anomaly detection method using variational autoencoder (VAE). In contrast to CAE which often uses the reconstruction error to detect anomalies, variational autoencoder (VAE) reason via the reconstruction probability. This allows for more principled and objective decisions (An and Cho, 2015).

Furthermore, many attempts aim to improve the performance of VAE including attribute-conditioned VAE (Yan et al., 2016). However, a high improvement was achieved by using diverse loss functions, considering the shortcomings of pixel-wise losses (Snell et al., 2017). Snell et al. (2017) proposed a structural similarity index (SSIM) between reconstructed and real data and demonstrated that human perceptual judgment is a better measure of image quality.

Hou et al. (2017) use a perceptual loss to encourage the VAE to learn a more meaning-full representation in the latent space. The same observation was made by Shvetsova et al. (2021), they used a CAE trained with a perceptual loss to detect anomalies in medical images.

In our study, we detect anomalous grapevine berries in images utilizing a VAE with a feature perceptual loss (FPL). Since image data from healthy plants are much easier to acquire than image data from damaged berries in their full variability, we present an approach that learns only with healthy berries and does not require labels from damaged berries. Specifically, our contributions are:

- The formulation of a VAE that is trained with a perceptual loss using only images of healthy plant material to capture the characteristics of healthy plants and identify anomalous patterns of damage and diseases.
- A framework that can identify anomalous patterns in images of grapevine in the field.
- A visualization of anomalies with heatmaps indicating diseased and damaged areas.

## 2. Materials and Methods

We use a dataset that was acquired in the field at the Julius Kühn-Institut Geilweilerhof located in Siebeldingen, Germany (49°21.7470 N, 8°04.6780 E) containing images of grapevine plants taken with a field phenotyping platform.

### 2.1. Sensor System

The field phenotyping platform called Phenoliner (Kicherer et al., 2017) consists of a modified grapevine harvester from ERO Gerätebau (Niderkumbd, Germany), namely the ERO-Grapeliner SF200. After the removal of the harvesting equipment, including the shaking unit and destemmer, a camera system with artificial lightning and a diffuse background was installed in the “tunnel”. The camera is a red green blue (RGB) camera (DALSA Genie NanoC2590, Teledyne DALSA Inc., Waterloo, ON, Canada) with a 5.1-megapixel sensor and a 12 mm lens, where each image has a size of 2, 592 × 2, 048 pixels. Due to the restricted space inside the tunnel, we have an approximate distance of 0.75 m between camera and plant, leading to a real world resolution of 0.3 mm.

### 2.2. Data

We observed plants trained in the Vertical Shoot Positioned (VSP) system with the Phenoliner. The main characteristic of this training system is that only one main branch remains over the years, the rest of the canopy regrowth each season. The main berry region is at the lower part of the canopy and many leaves are removed to ensure optimal growth conditions. Furthermore, different varieties, namely Riesling, Felicia, and Regent, were observed during different growth stages, called Biologische Bundesanstalt, Bundessortenamt und Chemische Industrie (BBCH) stages. These stages include the stages BBCH75, with pea sized berries, and BBCH89, which is shortly before harvest.

**Figure 1:**
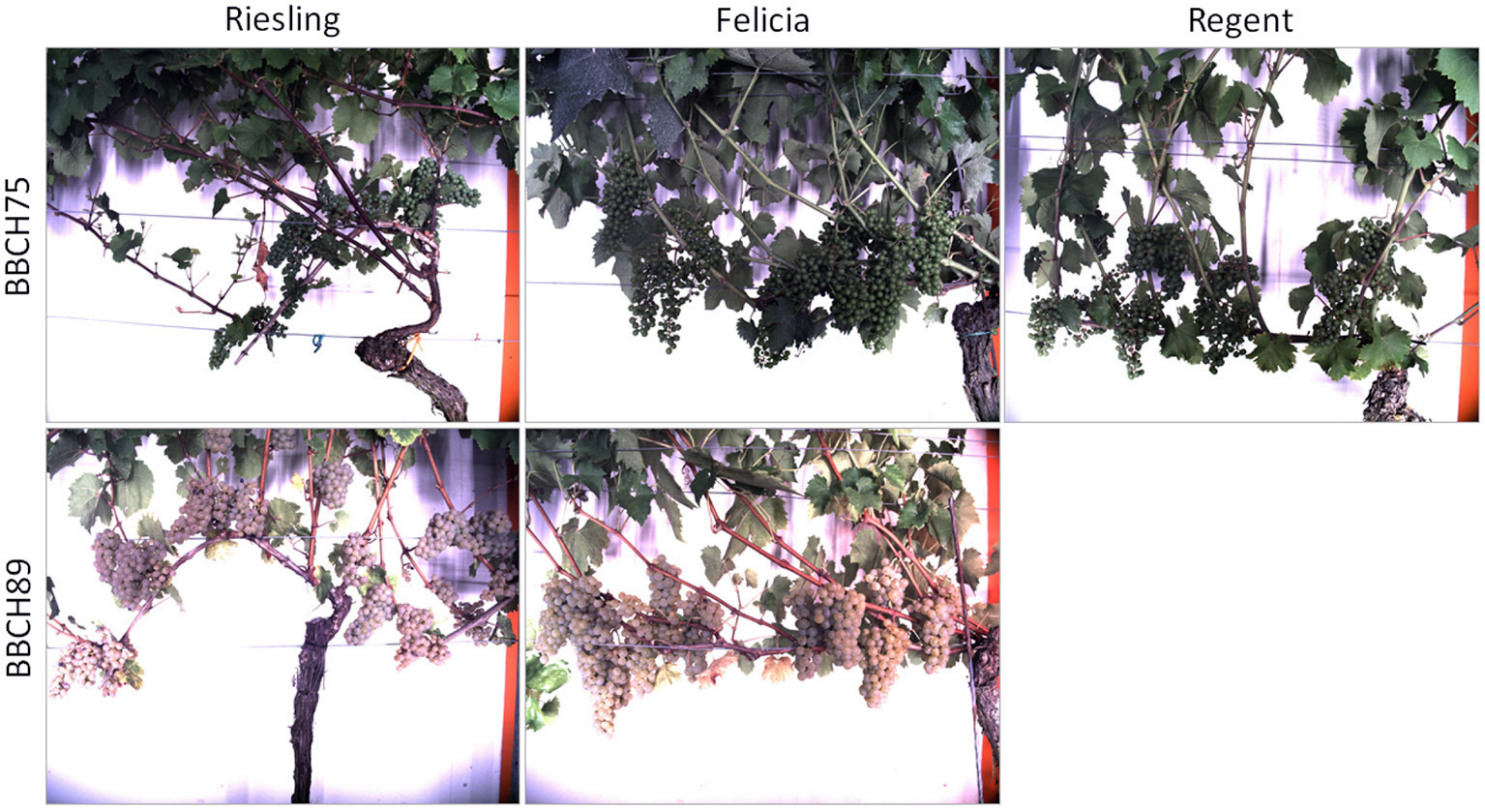
Examples from the dataset consisting of healthy plants without damaged berries, including different varieties and growth stages.

We collected 616 images of healthy plants (see Table 1), showing the lower part of the canopy where most of the berries are located. For the red variety Regent, we only selected images at the early BBCH stage, since we focus on green berries in this study. The berries change their color at a later stage, during the veraison. All observed plants were healthy and show no damaged berry regions. Examples from the training dataset can be seen in Figure 1.

**Table 1:** Overview of images showing healthy plants.

**Variety**	**BBCH75**	**BBCH89**	**Sum**
Regent	200	0	200
Felicia	76	79	155
Riesling	156	105	261

**Figure 2:**
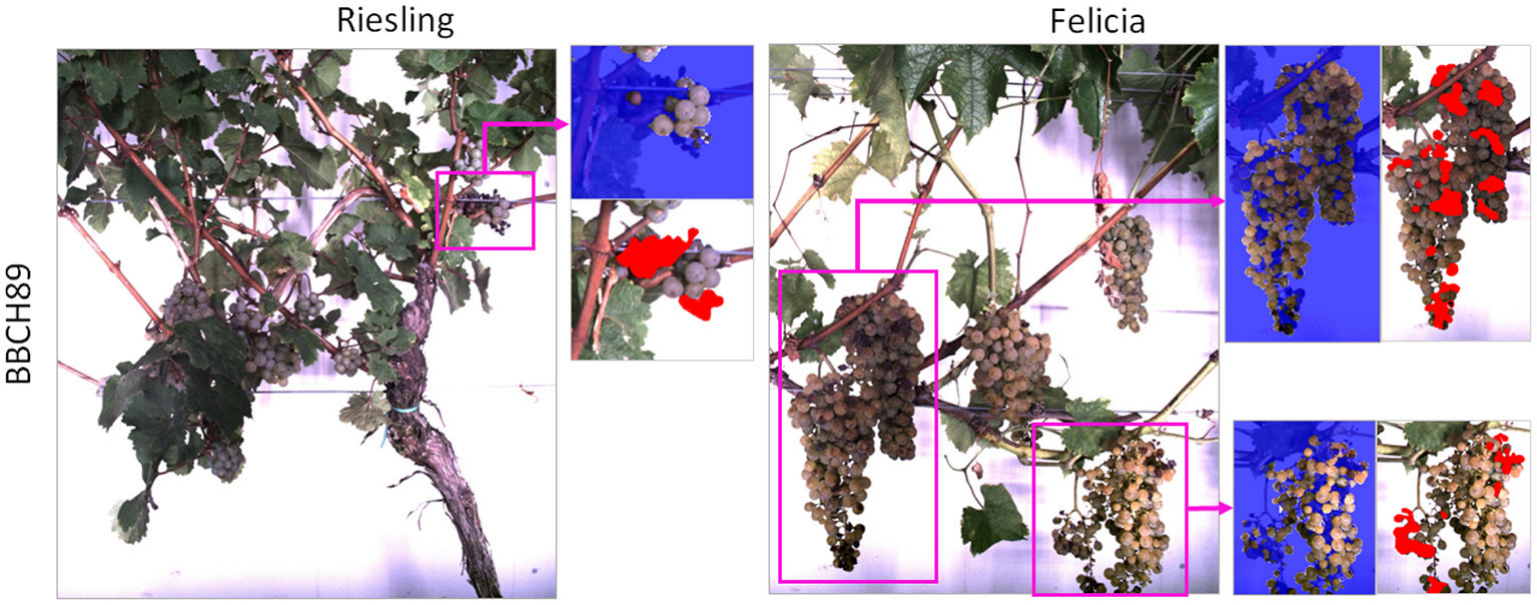
Examples from the images showing damaged grapevine berries. Damaged regions are highlighted with pink boxes. The blue masks in the image patches are the result of the pre-processing step of our proposed pipeline (refer to Section 2.3). The red areas in the patches are the manual damage annotations.

Additional 46 images showing damaged or diseased grapes (see Table 2) were selected for evaluation purposes. Since many damages occur during later stages and diseases develop during the season, we selected more images from the later BBCH stage. Two examples can be seen in Figure 2, the damaged grape regions are highlighted. The damages range from color variations to fully withered berries. For the images showing damaged grapevine berries, we provide manual annotations of the damaged regions.

**Table 2:** Overview of images showing damaged berries.

**Variety**	**BBCH75**	**BBCH89**	**Sum**
Regent	6	0	6
Felicia	1	24	25
Riesling	3	12	15

### 2.3. Pipeline

**Figure 3:**
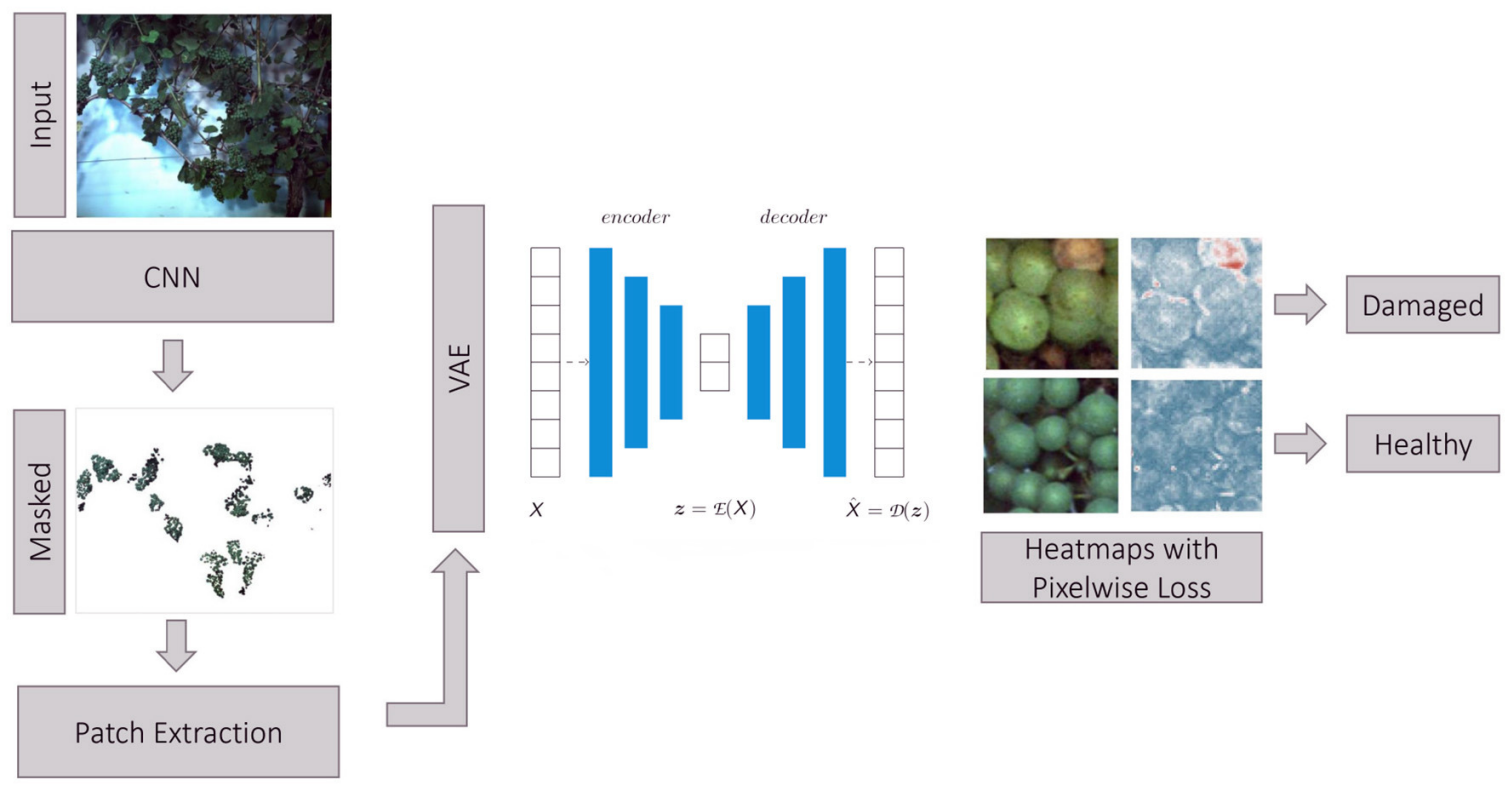
Proposed pipeline for the identification of anomalous grapevine berries using a VAE. Input images are segmented using a CNN (Zabawa et al., 2020) classifier which provides a semantic segmentation of berries and background. The resulting segmentation masks represent regions of interest containing bunches of berries. In these regions, we extract patches and feed them into a VAE. The deviation between reconstructed and input image is measured pixel-wise using a defined metric and used to calculate heatmaps highlighting anomalous image regions considered as damaged berries.

We propose a full pipeline for the detection of anomalous grapevine berries in images. As a pre-processing step, we use a CNN classifier to yield a semantic segmentation mask of grapevine berries and background with the goal to identify image regions containing bunches of berries (so-called regions of interest). For more details regarding this network architecture and performance, we refer the reader to Zabawa et al. (2020). Hereby, we assume, that anomalous berries occur mainly in close vicinity to healthy ones and the pre-processing result can be used to discard a majority of background information that is not in the focus of our application. For further analysis, 130 × 130 image patches are extracted from the original image within the region of interests. We chose this patch size to ensure that a certain number of berries is visible in each patch. This is the case for a patch size of 130 pixels leading to around 6 to 10 berries in each patch. Furthermore, the extracted patches are non-overlapping. Before the patches are fed into the VAE, the patches are resized to 64 × 64 pixels to enable faster image processing. The core of our pipeline is a VAE, which operates on the extracted image patches. A detailed description of the VAE is presented in Section 2.3.1. As a final step, we compute heatmaps highlighting areas with damaged grapevine berries. An overview of the whole proposed pipeline can be seen in Figure 3.

#### 2.3.1. Variational Autoencoder

The core of our network is a VAE, a neural network that performs a stochastic mapping of an input image **I** ∈ ℝ^*H,W,C*^ to a latent representation of smaller dimension $\text{Z}\in {\mathbb{R}}^{Z_{h},Z_{w},Z_{c}}$, also called bottleneck layer, and back to the output image $\hat{\text{I}}\in {\mathbb{R}}^{H,W,C}$ with the same dimension as the input. Since we use image data, all our variables are third order tensors, representing the height *H*, width *W*, and channels *C* of a single image. The mapping is done by a multi-layer AE (Hinton and Salakhutdinov, 2006) reformulated in a probabilistic fashion. This has been presented by the VAE implementation (Kingma and Welling, 2013) consisting of an encoder network E and a decoder network D (Schmidhuber, 2015). While the encoder E is able to embed an input image *X* in a latent representation z, the decoder D restores the original data by retaining the initial information. The low-dimensional embedding can be formulated as finding the best encoder/decoder pair.


(1) $$\begin{array}{l} {\text{E}}^{*},{\text{D}}^{*}={\mathrm{argmin}}_{\text{E},\text{D}}={\text{L}}_{\text{rec}}\left(\text{X}-\text{D}\left(\text{E}\left(\text{X}\right)\right)\right), \end{array}$$

where ${\text{L}}_{\text{rec}}$(.) is an error function defining the reconstruction error between the real and the reconstructed data. During training, a VAE aims to optimize the marginal log-likelihood of each observation (pixel) in dataset *X*. The VAE reconstruction loss ${\text{L}}_{\text{rec}}$ is the negative expected log-likelihood of the observations in *X*:


(2) $$\begin{array}{l} {\text{L}}_{\text{rec}}=-E_{q\left(z|\text{X}\right)}\left[logp\left(\text{X}|z\right)\right]. \end{array}$$

In addition to the reconstruction error, an additional property of a VAE is the conditional distribution of z. The distribution *q*(z|*X*) of the latent vector z is given using the encoder network E, *q*(z|*X*): = E(*X*, ϵ). Here, ϵ is an auxiliary noise variable ϵ~N(0,1) used to control the probabilistic distribution of z. The distribution of the latent vector z is enforced to be an independent random variable following a Gaussian normal distribution z~N(0,1). The difference between the *q*(z|*X*) and N(0,1) is quantified by using the Kullback-Leibler (KL)-Divergence:


(3) $${\text{L}}_{\text{KL}}={\text{D}}_{\text{KL}}\left(q\left(\text{z}|X\right)||N(0,1)\right)$$

A reparameterization trick is used to sample from the domain of latent vectors Z, allowing direct backpropagation (Kingma and Welling, 2013). The VAE is trained by simultaneously optimizing the reconstruction loss ($L_{\text{rec}}$) and the KL-Divergence ($L_{\text{KL}}$):


(4) $$\begin{array}{l} {\text{L}}_{VAE}={\text{L}}_{rec}+{\text{L}}_{\text{KL}} \end{array}$$

For more detailed information, we refer the reader to Kingma and Welling (2013).

**Figure 4:**
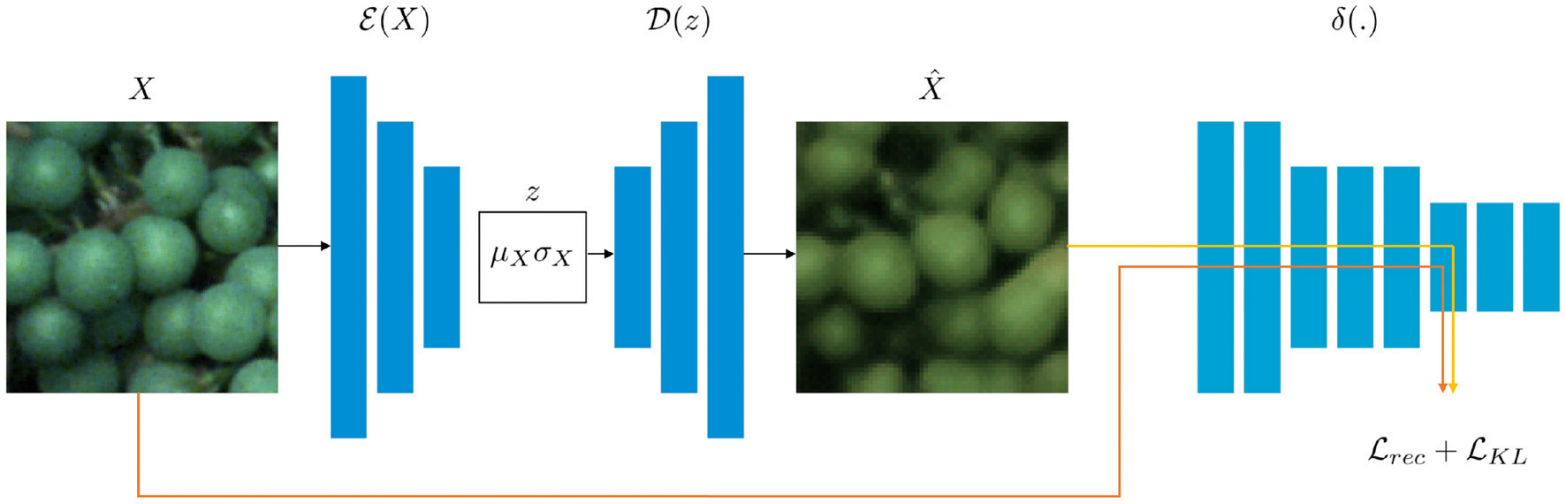
The architecture of our VAE is realized by two neural networks (Hinton and Salakhutdinov, 2006), namely an encoder network E, and a decoder network D. While D embeds the input data *X* with dimensionality *M* in a latent representation Z of dimensionality *m* (where *m*≪*M*), D is able to restore the data $\hat{X}$ given the latent representation *z*. Both *X* and $\hat{X}$ are fed into a VGGNet (Simonyan and Zisserman, 2014) to calculate the reconstruction loss ${\text{L}}_{\text{rec}}$. In addition, a KL-Divergence loss ${\text{L}}_{\text{KL}}$ is calculated.

Our overall approach is based on the framework proposed by Hou et al. (2017) containing three main sub-networks, namely an encoder network E, a decoder network D, and a pre-trained CNN δ which is used to calculate the loss function in deep feature space (refer to Figure 4). The pre-trained CNN δ is a network from the Visual Geometry Group (VGG), called a VGGNet (Simonyan and Zisserman, 2014), to compare the hidden layer representations by measuring the difference, termed as FPL $L_{\text{rec}}$ between input image *X* and the reconstructed image $\hat{\text{X}}$. We only update E and D during training while fixing δ. In addition, a KL-Divergence loss $L_{\text{KL}}$ is used to ensure that the latent vector z is an independent random variable.

#### 2.3.2. Feature Perceptual Loss

Instead of comparing the input image with the reconstructed output image by using a pixel-wise loss, we feed both the input image and the reconstructed image into a pre-trained CNN (Simonyan and Zisserman, 2014) to measure the difference between the hidden layer representations with an FPL. The measured FPL is intended to report important perceptual quality features and small differences in the hidden representation and, thus, can provide a better quality of the reconstructed image compared to pixel-wise losses (Hou et al., 2017). The FPL loss is defined as ${\text{L}}_{\text{rec}}=L^{1}+L^{2}+L^{3}+...+L^{l}$, where $L^{l}$ is the feature loss at the *l*^th^ hidden layer. At the *l*^th^ layer, the representation of the input image *X* is given by δ(*X*)^*l*^ with *l*^th^ ∈ ℝ^*H*^^*l*^,*W*^*l*^,*C*^*l*^. Here, *C*^*l*^ represents the number of filters in the pre-trained CNN at the *l*^th^ layer with width *W*^*l*^ and height *H*^*l*^. We define the FPL ${\text{L}}_{\text{rec}}^{l}$ at the *l*^th^ layer between the input image *X* and the reconstructed image $\hat{\text{X}}$ by the squared Euclidean distance in each channel:


(5) $$\begin{array}{l} L_{\text{rec}}^{l}=\frac{1}{2C^{l}W^{l}H^{l}}=\overset{C^{l}}{\underset{c=1}{\sum }}\overset{W^{l}}{\underset{w=1}{\sum }}\overset{H^{l}}{\underset{h=1}{\sum }}\left(\delta {\left(\text{X}\right)}_{c,h,w}^{l}-\delta {\left(\text{X}\right)}_{c,h,w}^{l}\right). \end{array}$$

The total loss of overall hidden layers is given as the sum of different layers in the CNN network:


(6) $$\begin{array}{l} {\text{L}}_{\text{rec}}=\underset{l}{\sum }{\text{L}}_{\text{rec}}^{l}. \end{array}$$

The final objective includes a KL-Divergence loss, leading to the following loss function:


(7) $$\begin{array}{l} \text{L}=\alpha {\text{L}}_{\text{KL}}+\lambda {\text{L}}_{\text{rec}}, \end{array}$$

where the weighting between both loss terms is controlled with the hyper-parameters α and λ.

### 2.4. Experimental Setup

#### 2.4.1. Training Details

We train our pre-processing network with the publicly available berry segmentation dataset presented in Zabawa and Kicherer (2021). Our AEs are trained on patches of healthy berries which have an original size of 130 × 130 and which are resized to 64 × 64 pixels, showing a good compromise between computation time and accuracy. The patch extraction process was described in detail in Section 2.3. Our dataset contains a total size of 5,041 patches, where the patches show healthy berries of all varieties, namely Riesling, Felicia, and Regent of different BBCH stages. We create a balanced data set with respect to the variety and growth stage. This is important since the expressed color is a major determining factor and should be properly learned by a model. From the healthy patches, we split 20% for the test set. We add 858 patches from the images showing damaged grapevine berries, resulting in a balanced test set containing 1,866 patches from healthy and damaged berries. We train with a batch size of 64 using the Adam optimization algorithm as a respective optimizer (Kingma and Ba, 2014). We use early stopping to avoid over-fitting with an initial learning rate of 0.0005. For validation, a batch size of 16 is used. These initial settings are used for all trained models to allow a fair comparison.

#### 2.4.2. Architecture

First, we briefly present the network architecture of the CNN, which we use to identify the regions of interest. Then, we describe the AEs, which were investigated in detail in our study, including our proposed VAE and an AE. The latter is presented to analyze the potential of our method in comparison to existing methods.

**CNN**: The semantic segmentation network has a classical U-shaped encoder-decoder structure. The encoder backbone is a MobileNetV2 (Sandler et al., 2018), and the decoder used is the DeepLabV3+ (Chen et al., 2018). The combination of encoder and decoder results in a fully convolutional semantic segmentation network. The framework is based on an open-source implementation by Milioto et al. (2019) and was successfully used for berry segmentation by Zabawa et al. (2020). For more details about the motivation of the design choices and training details, we refer the reader to Zabawa et al. (2020).

**VAE:** We based our VAE on the architecture which was proposed by Hou et al. (2017). The encoder consists of 4 convolutional layers, each with 4 × 4 kernels. To achieve spatial downsampling, a stride of 2 is chosen instead of a pooling operation. After each convolutional layer, we apply a batch normalization and Leaky Rectified Linear Unit (ReLU) activation layer. The center part of the VAE features two fully connected layers which are used to compute the KL-divergence loss. The two fully connected layers represent the mean and variance. The decoder also consists of 4 convolutional layers, but with 3 × 3 kernels and a stride of 1, and replication padding. To ensure that the output and input have the same resolution, upsampling is performed using the nearest neighbor method with a scale of 2. To stabilize the training, batch normalization and LeakyReLU activation are applied as well. We use the 19-layer VGGNet (Simonyan and Zisserman, 2014) to compute the FPL. In the following, we refer to our network as FPL-VAE, for more information, we refer the reader to Hou et al. (2017).

**AE:** As a baseline architecture, we use an AE inspired by Strothmann et al. (2019). We use four convolutional encoder and decoder layers, each with 3 × 3 kernels, padding of 1, and a stride of 1. We use LeakyReLU as an activation function. After each convolutional layer, we apply batch normalization. We test two types of loss functions. We first analyze an SSIM (Wang et al., 2004). We refer to this network as SSIM-AE. Following our approach, we analyze the same network architecture but use the same 19-layer VGGNet (Simonyan and Zisserman, 2014) to compute an FPL. We refer to this network as FPL-AE.

## 3. Results

In the following, we will discuss the main experiments which were conducted in this study. In the first one, we explore the general application of our method to detect anomalies in grapevine berries. We extensively evaluate the results on the available dataset, first with the image-wise metric for all used species, and second with the pixel-wise heatmaps. The experiments are based on the field-based grapevine dataset and explore the potential of the automatic heatmap generation for the identification of damaged grapevine berries.

### 3.1. Qualitative Image Reconstruction

**Figure 5:**
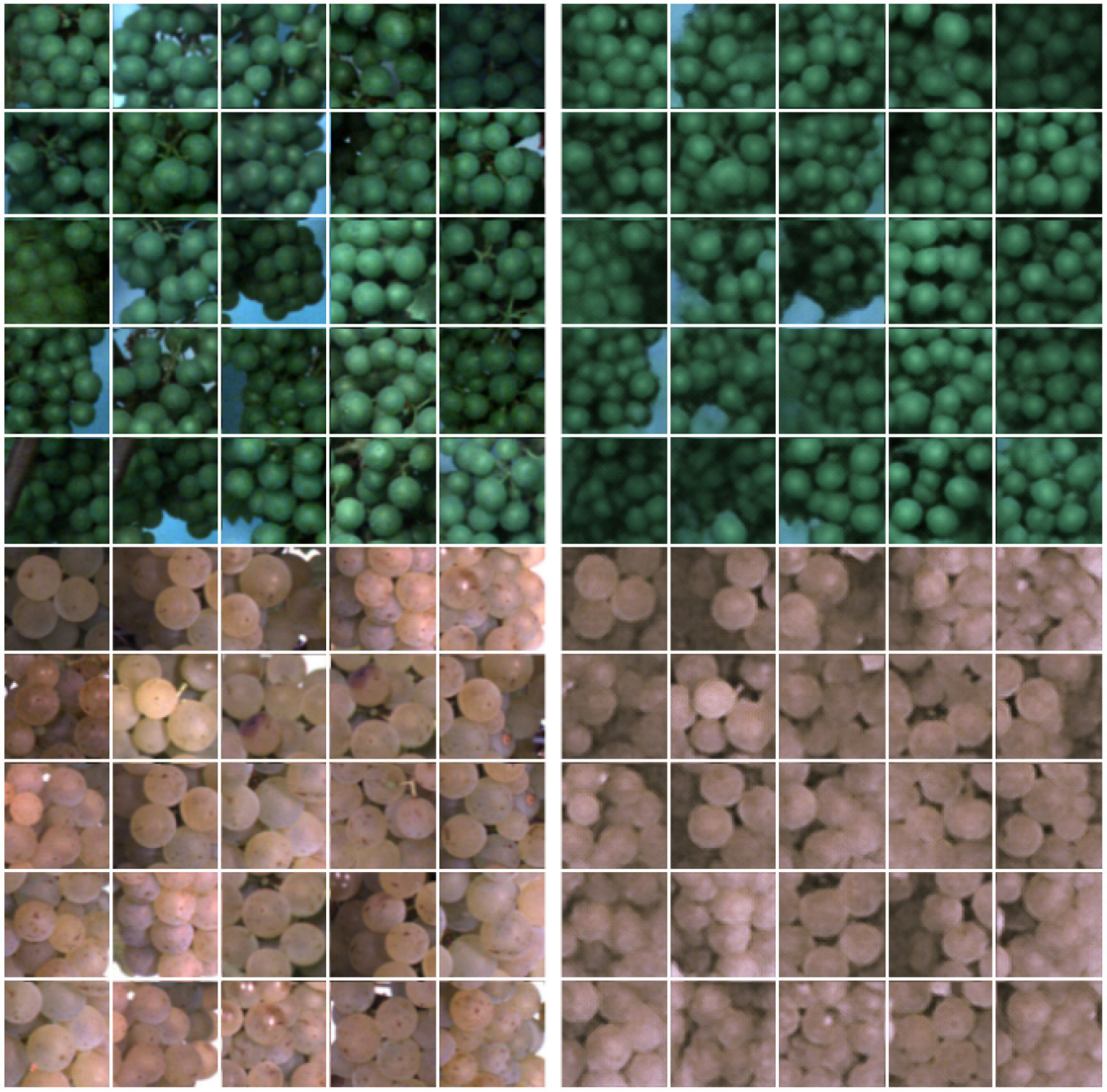
Images and image reconstructions of non-anomalous grapevine image patches using our proposed FPL-VAE. The original image patches are displayed on the left side, and the corresponding reconstructions are displayed on the right. The upper patches show berries in the BBCH75 stage, and the lower ones shortly before harvest in the BBCH89 stage.

Figure 5 illustrates example results for image reconstructions using our proposed FPL-VAE model. We show examples of two different varieties from two different phenotypic stages. The images show that the colors are well preserved independent of the grapevine color. In addition, the shapes are faithful to the reference image. This is especially apparent in the upper patches, where the background is visible. Although we use a stochastic model, reconstructions are similar to the input regarding the number and shape of berries as well as the position and color. However, we notice that the reconstructed images appear to be slightly more blurred compared to the original images.

### 3.2. Loss Functions and Architectures

In this section, we compare our proposed FPL-VAE with an AE which was proposed by Strothmann et al. (2019) and successfully applied to anomaly detection in grapevine. We investigate the model accuracy with respect to the loss function which is used to train the networks and explore which loss is best suited to detect anomalies. In detail, we investigate the (ℓ1), the mean squared error (MSE), and the binary cross entropy (BCE) losses for the detection of anomalies.

**Figure 6:**
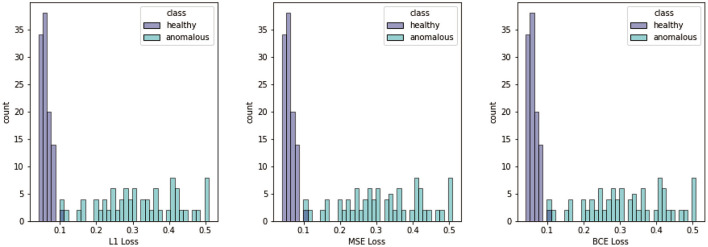
Histograms of different losses, namely ℓ1, MSE, and BCE. Losses are computed for all test patches containing healthy and anomalous berries.

In Figure 6, we display the summed loss scores for the test data calculated with the different metrics. The figure shows that we have a significant difference between images showing healthy and anomalous berries for all metrics. In all cases, the loss takes mainly small values for the patches showing healthy berries. Therefore, the histogram for this class is narrow. In contrast to this, the loss obtained for patches with anomalous berries takes a wide variety of values. The histogram is broad and has no clearly identifiable maximum.

In Table 3, we show the detection accuracy of anomalous grapevine berries for three different network architectures and three different losses. The accuracy is based on the loss score. An iterative optimization technique is used to find the best threshold for all approaches. The threshold is used to decide about the damaged state of the berries, and the results are used to calculate the accuracy. The table shows that our proposed FPL-VAE outperforms the other two networks. Especially the combination of the FPL-VAE and the ℓ1 loss yield the best result. Only for the BCE-loss does the FPL-AE performs better than our FPL-VAE.

**Table 3:** Model accuracy for anomaly detection based on different loss maps and network structures.

	**L1 Loss**	**MSE Loss**	**BCE Loss**
SSIM-AE	0.859	0.852	0.856
FPL-AE	0.860	0.869	0.901
**FPL-VAE**	**0.923**	0.914	0.883

*The bold number indicates the network yielding the best result*.

### 3.3. Training Systems

We further analyze the model performance concerning the different BBCH growing stages. We restrict the experiment to the evaluation of the best performing model, namely the FPL-VAE model using an ℓ1 loss term.

Table 4 shows that the accuracy for the later growth stage is higher compared to the earlier one. However, the damage expressed in BBCH89 is more severe than in BBCH75 resulting in a not entirely fair comparison.

**Table 4:** Model accuracy for different BBCH stages.

	**Joint**	**BBCH75**	**BBCH89**
FPL-VAE	0.923	0.903	0.938

### 3.4. Anomaly Detection With Heatmaps

We use the proposed FPL-VAE model to detect anomalous grapevine berries. In addition, we suggest heatmaps to highlight anomalous regions within the reconstructed image. We measure the pixel-wise difference and propose an MSE to be the best metric for our use case since anomalous regions are penalized more compared to non-anomalous regions by taking the pixel-wise squared error.

**Figure 7:**
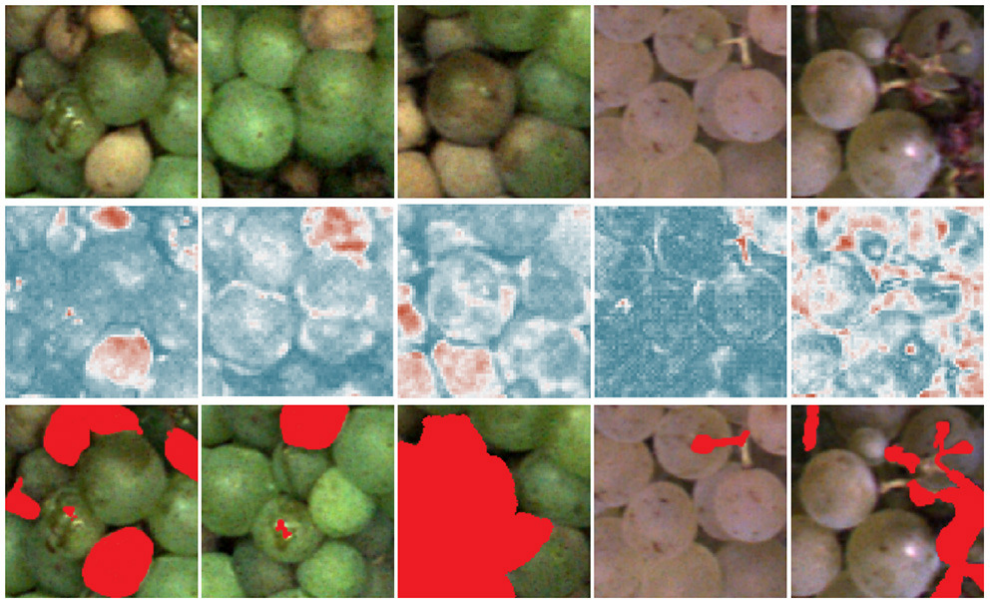
Results for pixel-wise anomaly detection obtained by an FPL-VAE for damaged patches. The original images are displayed in the upper row. A corresponding heatmap of loss values is displayed in the middle, red color represents anomalies, and dark blue indicates non-anomalies. The darker the color, the more certain the network is, that a berry is damaged or healthy. In the third row, the red color highlights the manual annotations of anomalous berry regions.

**Figure 8:**
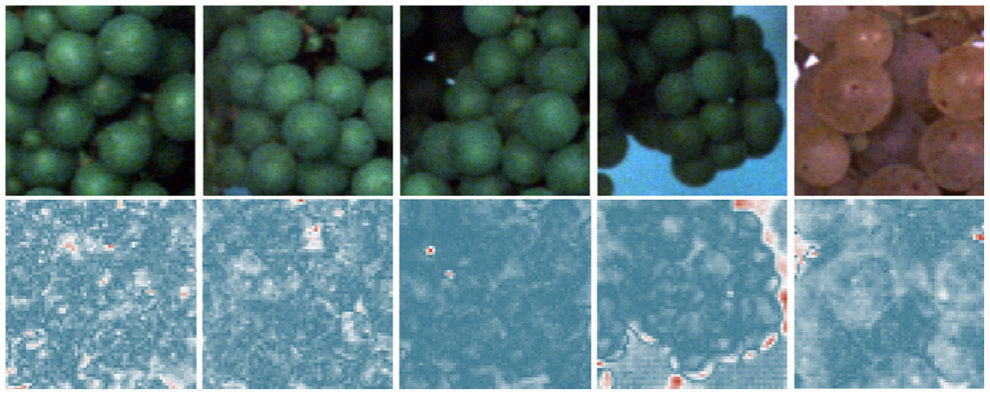
Results for pixel-wise anomaly detection obtained by an FPL-VAE for healthy patches. The original images are displayed in the upper row. A corresponding heatmap of loss values is displayed in the lower row, where a red color represents anomalies and dark blue indicates non-anomalies.

Figures 7, 8 show the heatmaps obtained from the pixel-wise MSE between reconstructed and input images which are used to detect anomalous grapevine regions. The images in Figure 7 exhibit varying degrees of damage and thus underline the potential of the proposed framework to detect the most infected regions. The results comprise different varieties at different BBCH stages and various types of defects such as berry rot, sun burn, atrophy, or malformation. Not all damages are detected with the same confidence, which is especially apparent in the middle patch. The big grape in the center is not detected with high confidence, but the overall patch is correctly identified as damaged. On the other hand, even a small anomaly, like the small stem in the second example from the left, is highlighted in the heatmap.

We also provide examples of heatmaps for healthy image data in Figure 8. All loss maps show high confidence that the patch is healthy. Only in the 4th image is the border area between the berries and background falsely marked as an anomalous region. This indicates a drawback of the proposed framework. At border regions, the reconstruction will be inaccurate and, therefore, may result in false positive detections.

**Figure 9:**
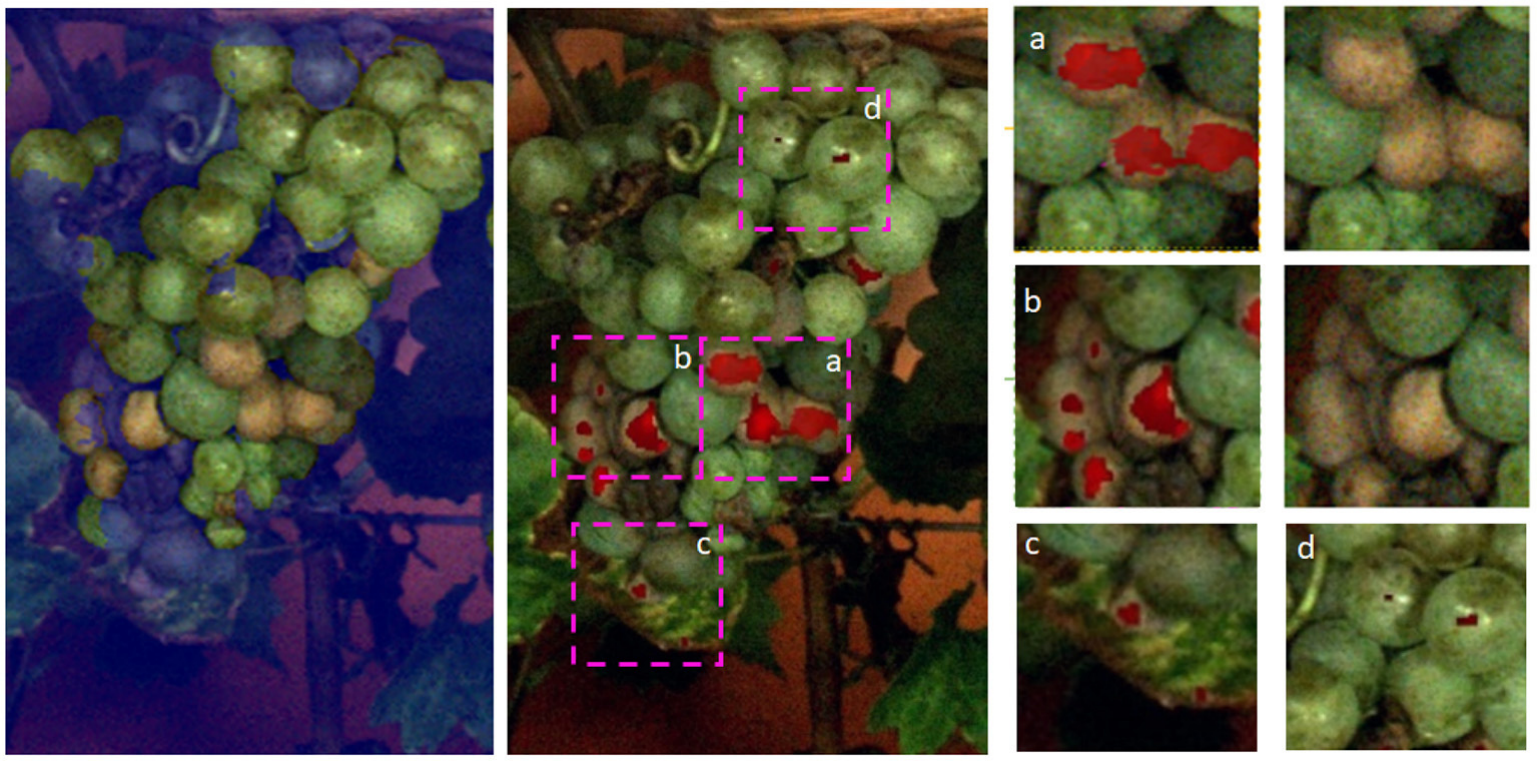
Results for a whole grape bunch. On the left, the applied mask can be seen, which is provided by the CNN. The mask is not perfect but as assumed the damaged areas are in close vicinity to healthy berries. In the middle, we see the predictions of our FPL-VAE in red. we extracted patches and show them in detail on the right side.

We further show the first results for heatmaps on the grape bunch level (refer to Figure 9). We can see that most areas containing damaged berries are correctly identified by our proposed method. Good examples are presented in the extracted patches a and b in Figure 9. In patch c we can also see, that a damaged leaf region was detected. In patch d on the other hand, we can see that light reflections were falsely detected as anomalies. Overall, the results show that the proposed method is able to distinguish between healthy and anomalous berries and to detect small and large anomalous regions, underlining the broad applicability of the approach.

## 4. Discussion

We trained our network on images showing grapevine plants. The images were acquired under real world conditions in the field using a phenotyping platform. Since it is challenging to evaluate the quality of reconstructed images objectively, we show several examples of patches showing healthy berries, which are determined by our FPL-VAE (refer to Figure 5). Although the network was trained on different varieties, at different BBCH stages under varying illumination, we showed that the reconstructions preserve the characteristics of each group. This includes the berry color and number, the shape of the whole area as well as single grapes, and the occurring background and lightning differences. The reconstructed images only appear slightly more blurry in comparison with the original image.

The next step went from the reconstructed patches to the differentiation of patches showing healthy and damaged berries with an iteratively optimized threshold of the loss values. We showed the histograms for the two different classes with respect to the different loss maps. All losses showed promising results for the classification between healthy and damaged patches. The histograms for the healthy class were narrow with a clear maximum, indicating that a healthy phenotype can be learned successfully. The histograms for the anomalous class on the other hand show large variations in the loss values and no clear maximum. This wide variation of loss values is in line with various degrees of damage, which are apparent in the patches, as well as a highly inhomogeneous appearance depending on the damage cause.

We also compare our proposed FPL-VAE network with an AE proposed by Strothmann et al. (2019), which was successfully applied to anomaly detection in grapevine. Furthermore, we also include an FPL into the AE to ensure a fair comparison of our approach. We showed that our network outperformed the other two, with 92.3% model accuracy compared to 90.1% for the best AE network. Furthermore, we can see an increase in model performance for the AE when the FPL is used. This underlines the potential of an FPL in contrast to the widely used pixel-wise losses and is in line with the findings of Hou et al. (2017).

Furthermore, we evaluate the potential of generated heatmaps for the detection of anomalies. We show example patches with damaged (refer to Figure 7) and healthy berries (refer to Figure 8). For the healthy examples, the loss maps show mainly low values, only in the 4th example in Figure 8 does the heatmap indicate anomalous regions. This occurs mainly at the border between the grape bunch and the background. Here, the network struggles to perfectly align the reconstruction with the original image. This could be seen as a drawback of our proposed method. However, as we extract patches from regions containing berries, we deliberately encourage the network to focus on the reconstruction of berries. Since the false positive detection occurs only in a very thin area around the grape bunch, the incorporation of knowledge regarding grape edges could filter out comparable false positive detections. In Figure 7, on the other hand, we can see that most regions containing damaged berries are correctly identified. Even very small artifacts like the stem in the 4th example are highlighted in the heatmap and correspond well with the manual annotations which can be seen in the bottom row.

Figure 9 shows a whole bunch of grapes. We show the mask which was used to extract the regions of interest. The mask does not cover the whole grape, but our assumption is that damaged grapes appear mostly in close vicinity to healthy ones. This is supported by this example. We can see that in the middle of the grape bunch damaged berries were successfully identified (refer to Figure 9 patch a, b). Another interesting observation is that in Figure 9 c, an anomalous leaf area is also identified by our method. The leaf shows signs of Esca, a grapevine trunk disease. One of the most prominent symptoms of this disease is color changes on the leaves, starting with yellow-brown colors along the leaf veins. In Figure 9 d, we see small examples of false positive detections on berries with strong light reflections. The detected anomalies are only a few pixels large and could be prevented by discarding small singular detections, favoring larger areas. Overall we can see that most anomalous plant regions (including berries and a small portion of the leaf) can be correctly identified with our proposed method.

Currently, the application of our system is not possible in real-time. Nevertheless, the inference time per image is a few minutes, making a realistic near real-time application possible.

## 5. Conclusion

The constant monitoring of grapevine plants is a labor-intensive task that has to be performed by skilled experts with many years of experience. The importance of phenotyping perennial plants will become even more relevant in the next years due to climate change, which will introduce new diseases and challenges. Therefore, we propose an automatic and objective end-to-end method using VAE with an FPL to detect diseased and damaged berries, which can help to identify regions that require action or closer monitoring. One of the main advantages is that our network is trained only on image patches showing healthy plant material. We do not need to extensively annotate data on an object or even pixel level. We show the capability of our VAE to detect unhealthy berries in a real-world field dataset with complex structures collected with a phenotyping platform. Our approach is especially suited for practical use since it is easy and fast to adapt to new vineyards or varieties without time-consuming annotation work. Furthermore, the growing market for Unmanned Aerial Vehicles (UAVs) makes our approach also more relevant since data can be acquired easily and fast. This is especially interesting due to the rapid development of UAVs, enabling ground-sampling distances of approximately $1\frac{mm}{pix}$ (Gogoll et al., 2020; Weyler et al., 2022), which would be sufficient for the berry level detection. When large amounts of data are available, it is important to provide fast and reliant results, which can guide a human observer to areas that need more monitoring.

## Data Availability Statement

The raw data supporting the conclusions of this article will be made available by the authors, without undue reservation.

## Author Contributions

MM and LZ designed and conducted the analyses. RR and UR helped to initiate the work. RR and LS helped to co-design the experiments. LZ and AK contributed to the data preparation. MM, LZ, AK, LS, UR, and RR contributed to the writing of the manuscript. All authors contributed to the article and approved the submitted version.

## Funding

This study was partially funded by the Deutsche Forschungsgemeinschaft (DFG, German Research Foundation) under Germany's Excellence Strategy–EXC 2070–390732324. Moreover, the study is partially funded by the German Federal Ministry of Education and Research (BMBF) in the framework of the international future AI lab AI4EO–Artificial Intelligence for Earth Observation: Reasoning, Uncertainties, Ethics and Beyond (grant no. 01DD20001).

## Conflict of Interest

The authors declare that the research was conducted in the absence of any commercial or financial relationships that could be construed as a potential conflict of interest.

## Publisher's Note

All claims expressed in this article are solely those of the authors and do not necessarily represent those of their affiliated organizations, or those of the publisher, the editors and the reviewers. Any product that may be evaluated in this article, or claim that may be made by its manufacturer, is not guaranteed or endorsed by the publisher.
